# Could Ghrelin Expression Regulate Diastolic Cardiac Function in Type 2 Diabetic Obese Patients?

**DOI:** 10.1002/dmrr.70049

**Published:** 2025-05-05

**Authors:** Celestino Sardu, Nunzia D'Onofrio, Maria Consiglia Trotta, Maria Luisa Balestrieri, Giovanni Francesco Nicoletti, Giovanbattista D'Amico, Carlo Fumagalli, Carla Contaldi, Giuseppe Pacileo, Lucia Scisciola, Maddalena Nicoletti, Ludovica Vittoria Marfella, Matilde Sbriscia, Ferdinando Carlo Sasso, Giuseppe Signoriello, Giuseppe Paolisso, Raffaele Marfella

**Affiliations:** ^1^ Department of Advanced Medical and Surgical Sciences University of Campania “Luigi Vanvitelli” Naples Italy; ^2^ Department of Precision Medicine University of Campania “Luigi Vanvitelli” Naples Italy; ^3^ Department of Plastic Surgery University of Campania “Luigi Vanvitelli” Naples Italy; ^4^ Department of Cardiovascular Diseases and Heart Failure Unit Monaldi Hospital Naples Italy; ^5^ Scientific Direction IRCCS‐INRCA Ancona Italy; ^6^ Department of Experimental Medicine University of Campania “Luigi Vanvitelli” Naples Italy; ^7^ Department of Medicine University of Saint Camillus Rome Italy

**Keywords:** abdominal fat, abdominoplasty, ghrelin, MicroRNA, obesity, oxidative stress, SIRT1

## Abstract

**Aims:**

Adipose tissue expresses cytokines, sirtuin‐1 (SIRT1), and microRNAs (miRs), regulating left ventricle (LV)‐diastolic function (LV‐DF). Ghrelin could modulate these pathways in patients with type 2 diabetes mellitus (T2DM) and obesity. We investigated ghrelin expression in T2DM obese patients after abdominal fat excision, and in those with LV‐DF normalisation at 1 year of follow‐up.

**Materials and methods:**

Two‐hundred and two T2DM obese patients enroled for abdominoplastic surgery were divided into those with normal LV‐DF (group 1: E/E′ < 9, *n* 76) and those with altered LV‐DF: group 2 (9 < E/E′ < 14; *n* 96) and group 3 (E/E′ > 14, *n* 28).

**Results:**

Patients with LV‐diastolic dysfunction had over‐inflammation, lower SIRT1 and higher abdominal fat sodium‐glucose‐transporter‐two (SGLT2) expression (*p* < 0.05). They did not differ for ghrelin expression (*p* > 0.05). They evidenced different tissue/serum expression of miR‐21, miR‐92 and miR‐126 (*p* < 0.05). Group 2 versus group 1 over‐expressed tissue inflammatory markers and SGLT2 (*p* < 0.05), with higher extent in group 3 versus group 1 (*p* < 0.01) and versus group 2 (*p* < 0.025). SIRT1 was downregulated in group 2 versus group 1 (*p* < 0.05), and versus group 3 (*p* < 0.01). At the follow‐up end, patients with lower LV‐diastolic dysfunction had lower inflammation and SGLT2, and higher serum ghrelin (*p* < 0.05). They increased miR‐126, and reduced serum miR‐21 and miR‐92 expression. At the follow‐up end, 50 patients experienced LV‐DF normalisation, which was predicted by tissue miR‐126 (HR 1.344, CI 95% 1.126–1.937), and ghrelin (HR 1.123, CI 95% 1.016–1.310).

**Conclusions:**

In T2DM obese patients, abdominal fat excision could reduce inflammation, up‐regulating serum ghrelin and inducing miRs implied in LV‐DF normalisation at 1 year of follow‐up.

**Clinical research trial number:**

NCT05988346.

## Graphical abstract

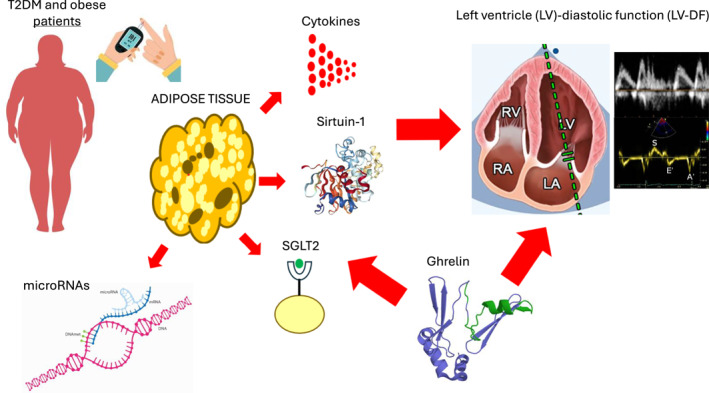

## 1 Introduction

Left ventricle diastolic dysfunction (LVDD) alters cardiac filling and causes fatigue, shortness of breath, dyspnoea, and heart failure (HF) [1]. LVDD could negatively affect the quality of life and worse clinical outcomes [1]. Obese patients, particularly those with type 2 diabetes mellitus (T2DM), have a higher rate of LVDD [2]. Indeed, overweight could alter cardiac structure and function by increasing preload and afterload and leading to left ventricle (LV) mass growth, LV dilation, and LVDD [2]. Obesity could alter glycaemic homoeostasis, worse insulin resistance, and T2DM status [3]. These responses increase the inflammatory burden and heart fibrosis, which further impair the LV diastolic mechanics via sirtuins down‐regulation [3, 4]. Notably, these pathways are also regulated by sodium‐glucose transporter 2 (SGLT2) [5], which increases inflammatory stress and down‐regulates sirtuins, leading to heart fibrosis and LVDD [6]. The inhibition of SGLT2 (SGLT2i) exerts anti‐inflammatory and cardio‐protective effects via sirtuins up‐regulation, leading to the best clinical outcomes [7, 8, 9]. SGLT2i shows pleiotropic effects on the cardiac pump [1, 10] and LV diastolic function [11]. Furthermore, T2DM obese patients under SGLT2i treatment exhibited an improved LV diastolic function via both glucose‐dependent and independent effects [12]. In this scenario, we evaluated ghrelin as a marker of insulin resistance, adipocyte metabolism, and weight loss [12], as well as a modulator of SGLT2 functions [13] and cardiac remodelling [14]. In animal models, ghrelin showed cardiovascular protective effects, rescuing sirtuin 1 (SIRT1) and reducing inflammatory/oxidative stress via microRNA‐126 (miR‐126) expression [14]. In T2DM patients with LVDD and HF, SGLT2i significantly reduced serum levels of miR‐21 and miR‐92, rescuing endothelial function and improving clinical outcomes through an amelioration of LV diastolic function [15]. Moreover, we hypothesised that these pathways could be modulated by ghrelin expression in T2DM obese patients with LVDD. Specifically, the abdominal fat surgical excision and weight loss could induce the over‐expression of serum ghrelin in T2DM obese patients. Thus, we first verified whether abdominal fat surgical excision and weight loss could induce serum ghrelin over‐expression, down‐regulate inflammatory burden, SGLT2 pathway, and rescue SIRT1 functions in obese T2DM patients. Secondly, we hypothesised that these effects might lead to the downregulation of miR‐21/miR‐92 and over‐expression of miR‐126 in obese T2DM patients. Here we investigated as our primary study hypothesis the difference in the proportion of subjects achieving a normalisation of LVDD between subjects starting with different stages of LVDD at baseline. We aimed to address these study hypotheses and to investigate the ghrelin‐induced consequences on the amelioration of LV diastolic function in T2DM obese patients. The effects of ghrelin on LV diastolic function in T2DM obese patients after surgical abdominal fat excision are not fully determined. Thus, to verify our hypotheses, we designed a prospective clinical study to evaluate the expression of inflammatory cytokines, SIRT1, SGLT2, miR‐21, miR‐92, miR‐126, and ghrelin in abdominal fat tissue biopsy at baseline, and the serum values of inflammatory cytokines, miR‐21, miR‐92, miR‐126 and ghrelin of obese T2DM patients without versus obese T2DM patients with LVDD at baseline and at 12 months of follow‐up after subcutaneous abdominal fat excision.

## 2 Materials and Methods

### 2.1 Research Design and Methods

In this observational study, conducted from January 2016 to January 2022, we prospectively screened consecutive obese patients with T2DM and standard indications to receive an abdominoplastic surgery [3]. We practiced the abdominoplasty for men and women desiring aesthetic improvement of the abdomen, body counting after excessive weight loss for women with significant skin and abdominal wall laxity following multiple pregnancies, and for bariatric patients who have excessive skin and/or pannus following significant weight loss [3]. Obesity was diagnosed as body mass index (BMI) > 30 [15]. All patients underwent abdominoplastic surgery and, after treatment, received a hypocaloric diet, with a mean recommended daily caloric intake of 1300 kcal, ranging from 1250 to 1350 kcal. The recommended composition of the dietary regimen was 55% carbohydrates, 30% lipid, and 15% protein. All enroled patients had a T2DM diagnosis according to international guidelines criteria [16]: fasting plasma glucose of ≥ 7.0 mmol/L (126 mg/dL; impaired fasting glucose [IFG]), 2‐h glucose of ≥ 11.1 mmol/L during a 75 g oral glucose tolerance test (GTT) (> 200 mg/dL; impaired glucose tolerance [IGT]), or plasma haemoglobin (Hb) A1c ≥ 48 mmol/mol (≥ 6.5%) [16]. To evaluate insulin sensitivity, we used the homoeostasis model to assess insulin resistance (HOMA‐IR) based on fasting plasma glucose (FPG) and insulin concentrations [16]. From the study population, we exclude the patients following these exclusion criteria: contraindication to receiving an abdominoplastic surgery; diagnosis of type 1 diabetes; T2DM in poor glycaemic control (HbA1c > 7%); patients without a confirmed diagnosis of T2DM; HF and coronary heart disease or depression of left ventricular ejection fraction (LVEF < 55%); uncontrolled blood pressure (blood pressure > 140/90 mmHg on two occasions 2 weeks apart); cardiac arrhythmias; severe anaemia, thyroid dysfunction, kidney failure; chronic neurological disorders; inflammatory chronic disease, and neoplastic disease. At the enrolment, all patients had normal results for laboratory data (urea nitrogen, creatinine, electrolytes, liver function tests, uric acid, thyroxin, and complete blood count), chest X‐rays, and electrocardiograms. The obese T2DM patients were evaluated at baseline and at 12‐month follow‐up. Echocardiography can furnish a reliable non‐invasive measurement of LV diastolic function [17]. We characterised LVDD based on the values of the ratio between the early (E) transmitral flow diastolic filling velocities and the tissue Doppler imaging (TDI) E wave (E′) [18]. We considered an E/E′ ratio >14 as a non‐invasive estimate of increased LV filling pressure [18]. E/E′ ratio could be used in the clinical setting to characterise the patients with versus those without LVDD, according to E/E′ cut‐off values [18]. Thus, our patients were divided into three groups: (i) patients without LVDD (E/E’ < 9; group 1) and (ii) those with LVDD (E/E′ > 9). Patients with LVDD were subdivided into two groups: group 2 (patients with 9 < E/E′ < 14), and group 3 (patients with E/E′ > 14) [18]. All study groups volunteered for repeated clinical evaluations, laboratory analyses, and echocardiography. In the follow‐up period, patients were managed by applying a multidisciplinary approach consisting of diet, exercise, and behavioural/nutritional counselling as described previously [3, 4]. The enroled patients were followed quarterly on an outpatient basis until 12 months. Each patient provided informed written consent to participate in this study. The Ethical committee of the participating institutions approved the study. The patients also subscribed to a separate informed consent to undergo surgical intervention of abdominoplasty.

### 2.2 Anthropometrics Parameters

In all enroled patients, we measured the BMI and Waist‐hip ratio (WHR), an index of central obesity defined as waist circumference in centimetres divided by hip circumference in centimetres [19]. We evaluated insulin blood levels, HOMA‐IR, and serum values of ghrelin. We calculated the HOMA‐IR with the formula: HOMA‐IR = ((glycaemia × insulinaemia)/22.5) [16]. See the full discussion in supplementary files.

### 2.3 Echocardiography and Evaluation of LV Diastolic Function

Experienced physicians, fully trained in cardiovascular ultrasound, performed in all enroled patients trans thoracic 2‐dimensional and Doppler echocardiography using a Vingmed Vivid E9 echocardiograph (General Electric Vingmed Ultrasound, Milwaukee, WI, USA). We obtained the images using a 3.5‐MHz transducer and digitally stored them in three cardiac cycles for analysis. Thus, we measured the LV posterior wall thickness and inter‐ventricular septal dimension at end‐diastole (LVPWd and IVSd, respectively) via a two‐dimensional ECHO‐guided M‐mode approach. We performed the exams at baseline and at 12 months to assess the LV diastolic function as recommended [18]. We evaluated LVDD by traditional Doppler and new TDI‐derived parameters, measuring the *trans*‐mitral flow parameters, including the early (E) and late (A) diastolic filling velocities, the E/A ratio, and the E deceleration time (DT) from an apical four‐chamber view with conventional pulsed wave Doppler [18]. We used the E/E′ ratio to characterise at baseline and follow‐up the patients without LVDD (group 1, E/E′ < 9) versus those with LVDD (group 2, 9 < E/E′ < 14; group 3, E/E′ > 14). For the full description of echocardiographic measurements (LVEF, LVDD, etc.) see the supplementary files.

### 2.4 Analyses of Blood Samples

We took serum samples from the peripheral brachial vein and stored them for cytokine levels at a temperature under 80°C until assayed. We determined the serum concentrations of tumour necrosis factor‐alpha (TNFα), interleukin 1 and 6 (IL1, IL6), and nitrotyrosine in triplicate using a highly sensitive quantitative sandwich enzyme assay (ELISA, Quantikine HS; R&D Systems, Minneapolis, MN). Assays for serum total and high‐density lipoprotein cholesterol, triglyceride, and glucose levels were performed in the chemistry laboratory of the hospital. We assayed the plasma insulin levels by radioimmunoassay (Ares, Serono, Italy), and assessed the insulin resistance in the fasting state using HOMA‐IR. We calculated the HOMA with the following formula: fasting plasma glucose (millimoles per litre) times fasting serum insulin (microunits per millilitre) divided by 25 as described [16]. We evaluated the levels of miRs and ghrelin from these venous samples. All these analyses were performed at baseline and repeated at 1 year of follow‐up in the study cohorts. Intervention of abdominal surgery and analysis of adipose tissue homogenates and protein extraction are reported in supplementary files.

### 2.5 Enzyme‐Linked Immunosorbent Assay (ELISA)

We evaluated SGLT2, Ghrelin, SIRT1, TNF‐α and IL‐6 expression levels in adipose abdominal superficial tissue samples from obese T2DM patients by ELISA (Quantikine HS; R&D Systems, Minneapolis, MN). Levels of Ghrelin, TNF‐α, and IL‐6 were evaluated at baseline and at 12 months in serum samples. See full description in supplementary files.

### 2.6 Analysis of miR‐21, miR‐92 and miR‐126 Expression

At baseline, we evaluated the adipose tissue expression of miR‐21, miR‐92 and miR‐126. The miR levels were also evaluated in serum samples at baseline and at 12‐month follow‐up. For the determination of adipose tissue miR‐21, miR‐92, and miR‐126 levels, the extraction, reverse transcription, and amplification of these miRs were performed by Real‐Time PCR in triplicate as previously reported [3, 4]. We assayed the serum levels of miR‐21, miR‐92, and miR‐126 as described [14, 15]. We analysed the data with the 2^‐ΔCt method.

### 2.7 Study Endpoints

As the primary study endpoint, we evaluated the rate of obese T2DM patients (those affected by LVDD) with normalisation of LV diastolic function at the end of the follow‐up period. Supporting Information Figure S1. The patients with normalisation of LV diastolic function showed values of E/E′ < 9 at the follow‐up end. Here, we evaluated the serum levels of ghrelin in the patients with normal LV diastolic function (those with E/E′ < 9) versus those with LVDD (those with E/E′ > 9). The values of ghrelin were specifically evaluated in the LVDD patients, which were then differentiated into the two cohorts of study: the patients with 9 < E/E′ < 14 (group 2) or with E/E′ > 14 (group 3) at the end of follow‐up. As a secondary study endpoint, we evaluated in these cohorts of patients (group 1 vs. group 2 vs. group 3) the values of serum inflammatory markers, SGLT2, miR‐21, miR‐92, and miR‐126. Supporting Information Figure S1. Finally, we correlated, via Cox regression analysis, the baseline characteristics of study cohorts and the fat tissue expression of cytokines, SIRT1, ghrelin, and miRs to the rate of patients with normalisation of LV diastolic function at the end of the follow‐up period (1 year).

### 2.8 Statistical Analysis

We prospectively collected the data from electronic medical records (EMR) in the clinical setting at the participants' Institutions. We used electronic systems for data capture, collection, and monitoring, with onsite and real‐time data entry; the data were then analysed. Data were presented as group means ± SD. To evaluate in comparison of the proportions of obese patients in the three groups, those without (group 1) versus those with LVDD (group 2 and group 3) who showed normalisation of LV diastolic function at the end of the follow‐up period, a test for comparison of proportions was performed, and the One‐way analysis of variance (ANOVA) was used to compare baseline data, followed by pair‐wise comparisons. We used multiple comparisons between groups (group 1 vs. group 2, group 2 vs. group 3, and group 1 vs. group 3 of patients) via the Bonferroni test. The presented data were representative of group 1, group 2 and group 3 of patients enroled in the study. To evaluate the predictors' factors of the study endpoint (normalisation of LV diastolic function) at 1 year of follow‐up, we used the Cox regression models adjusted for potential confounders (systolic arterial pressure, tissue expression of ghrelin expression, miR‐126, CRP, and SIRT1; SGLT2i therapy). We calculated a univariate analysis to examine the association between a single risk factor and the 12‐month study outcome. Then, we entered a multivariate model all the variables with a *p*‐value of less than 0.1, as calculated in the univariate analysis. In the multivariate model, a *p*‐value of less than 0.05 was considered statistically significant. For all independent predictors, 95% confidence intervals (CI) were calculated. Statistical significance was established at a *p*‐value < 0.05 for all the other analyses.

To calculate the study population sample size, we used a statistical power of 90%, with an alpha error of 5%, to detect the expected event (normalisation of diastolic function) at 1 year of follow‐up in the study cohorts. Thus, in the three cohorts of study, supposing a 30% expected average event proportion (the normalisation of LV diastolic function at follow‐up end: E/E′ < 9), with a significant difference between groups, we calculated a sample size of 204 patients as the study population. We used a calculation based on the proportion between 3 groups of patients corrected for Bonferroni.

Statistical analysis was performed using the SPSS software package for Windows 17.0 (SPSS Inc., Chicago Illinois).

## 3 Results

### 3.1 Baseline Findings

We screened a study population of 209 patients; of these subjects, five did not sign the informed study consent and were excluded from the current investigation. Thus, we enroled a study population of 204 obese patients with T2DM.

At baseline, the patients (group 1 vs. group 2 vs. group 3) did not differ (*p* > 0.05) in clinical characteristics, glucose homoeostasis, insulin resistance, and drug therapy (Table 1). In contrast, significant differences in LAV, E, E/A, serum inflammation values, and SGLT2 expression (*p* < 0.05) were found (Table 1). Furthermore, patients with LVDD (group 3 vs. group 1, and group 2 vs. group 1) had more inflammation and a higher serum expression of nitrotyrosine and SGLT2 (*p* < 0.05). Table 1.

**TABLE 1:** Clinical characteristics of the study population at baseline and at follow‐up end.

Study variables	Baseline			1 year of follow‐up		
	Group 1 (*n* 80)	Group 2 (*n* 96)	Group 3 (*n* 28)	Group 1 (*n* 118)	Group 2 (*n* 74)	Group 3 (*n* 12)
Clinical variables						
Age	42.5 ± 8.4	45.7 ± 8.7	46.1 ± 6.3	43.5 ± 8.7	46.7 ± 8.8	47.1 ± 7.1
Male (%)	22 (27.5)	30 (31.3)	8 (28.6)	34 (28.8)	22 (29.7)	4 (33.3)
BMI (kg/m^2^)	34.5 ± 4.04	34.2 ± 3.80	35.6 ± 5.45	32.7 ± 3.35^c^	32.8 ± 3.50^c^	32.8 ± 3.03^c^
Systolic arterial pressure (mmHg)	128.4 ± 10.1	129.7 ± 10.5	128.3 ± 13.3	127.5 ± 10.8	128.6 ± 10.1	128.7 ± 11.5
Diastolic arterial pressure (mmHg)	83.4 ± 8.9	85.8 ± 7.9	85.1 ± 8.5	81.3 ± 8.3	83.2 ± 7.6	82.4 ± 8.6
Heart rate (beats for minute)	76.1 ± 9.0	78.2 ± 8.5	79.7 ± 9.2	68.5 ± 9.5^c^	71.9 ± 9.2^c^	70.6 ± 8.4^c^
WHR	0.91 ± 0.007	0.91 ± 0.005	0.91 ± 0.006	0.88 ± 0.003^c^	0.89 ± 0.0.04^c^	0.88 ± 0.006^c^
HOMA‐IR	3.5 ± 0.98	3.7 ± 0.68	4.1 ± 1.3	2.9 ± 0.80^c^	3.1 ± 0.83^c^	3.3 ± 1.07^c^
Insulin (IU/mL)	37.7 ± 8.46	39.1 ± 9.31	42.9 ± 12.12	32.4 ± 7.35^c^	34.0 ± 8.09^c^	37.4 ± 10.54
Glucose (mmol/L)	6.10 ± 0.84	6.22 ± 0.74	6.14 ± 0.82	6.06 ± 0.69	6.12 ± 0.71	6.08 ± 0.70
Cholesterol (mmol/L)	4.67 ± 0.95	4.69 ± 0.89	4.74 ± 0.91	4.29 ± 0.78^c^	4.35 ± 0.80^c^	4.41 ± 0.83
HDL (mmol/L)	1.88 ± 0.41	1.85 ± 0.43	1.89 ± 0.40	1.91 ± 0.49	1.89 ± 0.52	1.91 ± 0.41
LDL (mmol/L)	3.16 ± 0.37	3.31 ± 0.38	3.14 ± 0.35	2.82 ± 0.63^c^	2.91 ± 0.57^c^	2.85 ± 0.52^c^
Triglycerides (mmol/L)	1.89 ± 0.44	1.85 ± 0.54	1.88 ± 0.60	1.81 ± 0.53^c^	1.80 ± 0.49^c^	1.82 ± 0.44
Creatinine (mmol/L)	98.6 ± 4.4	101.2 ± 3.5	100.9 ± 4.0	92.4 ± 3.8^c^	95.9 ± 3.1^c^	96.2 ± 3.5^c^
Hb1Ac (%)	6.4 ± 0.05	6.5 ± 0.06	6.6 ± 0.08	6.3 ± 0.06	6.4 ± 0.03	6.5 ± 0.06^c^
Biohumoral markers						
CRP (mmol/L)	0.79 ± 0.32	1.05 ± 0.22^a^	1.08 ± 0.37^a^	0.57 ± 0.06^c^	0.83 ± 0.03^a^ ^,^ ^c^	1.02 ± 0.09^a^ ^,^ ^b^
IL6 (pg/mL)	4.18 ± 0.43	4.36 ± 0.35^a^	4.71 ± 0.55^a^	3.89 ± 0.26^c^	4.08 ± 0.35^a^ ^,^ ^c^	4.43 ± 0.22^a^ ^,^ ^b^
TNFα (pg/mL)	7.44 ± 0.16	8.17 ± 0.25^a^	8.53 ± 0.28^a^	5.86 ± 0.22^c^	6.58 ± 0.38^a^ ^,^ ^c^	7.65 ± 0.41^a^ ^,^ ^b^ ^,^ ^c^
Nitrotyrosine (nmol/L)	4.39 ± 0.21	5.18 ± 0.31^a^	5.29 ± 0.36^a^	3.52 ± 0.63^c^	4.59 ± 0.61^a^ ^,^ ^c^	5.19 ± 0.45^a^ ^,^ ^b^
miR‐21 × 10^2^, A.U.	0.71 ± 0.12	2.67 ± 0.52^a^	3.51 ± 0.69^a^	0.45 ± 0.33^c^	2.16 ± 0.52^a^ ^,^ ^c^	3.06 ± 0.69^a^ ^,^ ^b^
miR‐92 × 10^2^, A.U.	0.66 ± 0.12	1.26 ± 0.35^a^	1.77 ± 0.25^a^	0.28 ± 0.06^c^	1.16 ± 0.04^a^ ^,^ ^c^	1.78 ± 0.10^a^ ^,^ ^b^
miR‐126 × 10^2^, A.U.	1.61 ± 0.25	0.50 ± 0.03^a^	0.69 ± 0.21^a^	3.23 ± 0.80^c^	1.69 ± 0.16^a^ ^,^ ^c^	0.95 ± 0.39^a^ ^,^ ^b^ ^,^ ^c^
SGLT2	1.03 ± 0.52	2.43 ± 0.38^a^	2.55 ± 0.72^a^	0.94 ± 0.42	1.57 ± 0.65^a^ ^,^ ^c^	2.13 ± 0.54^a^ ^,^ ^b^
Ghrelin	299.58 ± 81.64	284.92 ± 64.13	253.28 ± 59.94	1060.49 ± 354.79^c^	710.48 ± 294.22^a^ ^,^ ^c^	540.07 ± 84.12^a^ ^,^ ^b^ ^,^ ^c^
Echocardiographic parameters						
Intima‐media thickness	1.01 ± 0.15	1.02 ± 0.18	1.02 ± 0.12	0.84 ± 0.17^c^	0.83 ± 0.18^c^	0.87 ± 0.12^c^
LVTDd (mm)	55.4 ± 4.2	55.3 ± 3.7	56.2 ± 4.4	54.3 ± 4.0	53.9 ± 3.8	54.9 ± 4.3
LVTSd (mm)	32.5 ± 4.2	32.8 ± 4.8	34.5 ± 4.6	31.8 ± 3.2	32.7 ± 3.8	34.5 ± 4.5
LVEF (%)	56.2 ± 6.5	55.8 ± 6.4	55.7 ± 3.9	57.8 ± 6.6	57.1 ± 6.1	58.2 ± 3.9
LAD (mm)	39.1 ± 4.3	42.3 ± 5.9^a^	47.5 ± 5.4^a^ ^,^ ^b^	35.1 ± 4.3^c^	40.1 ± 6.1^a^ ^,^ ^c^	46.7 ± 5.6^a^ ^,^ ^b^
LAV (ml/mq)	28.9 ± 3.14	32.5 ± 3.15^a^	36.3 ± 1.7^a^ ^,^ ^b^	28.1 ± 3.05	31.4 ± 2.75^a^	36.0 ± 1.75^a^ ^,^ ^b^
Septum (mm)	12.2 ± 2.1	12.4 ± 2.0	12.8 ± 1.8	9.8 ± 1.8^c^	10.8 ± 2.1^a^ ^,^ ^c^	11.3 ± 1.7^a^ ^,^ ^b^ ^,^ ^c^
Posterior wall (mm)	10.1 ± 1.1	10.4 ± 1.2	10.5 ± 1.3	9.1 ± 1.0^c^	9.3 ± 1.2^c^	9.3 ± 1.4^c^
LV mass (g)	208.7 ± 73.4	185.4 ± 58.6	199.6 ± 71.8	143.1 ± 43.8^c^	148.3 ± 42.3^c^	161.9 ± 52.8
LV mass/BSA (g/m^2^)	95.54 ± 34.9	89.50 ± 30.2	95.92 ± 37.7	66.31 ± 21.8^c^	70.89 ± 21.1^c^	82.03 ± 30.5
DecE‐time (m/s)	186.95 ± 26.75	233.37 ± 60.10^a^	126.64 ± 26.53^a^ ^,^ ^b^	183.10 ± 41.13	214.41 ± 65.77	130.14 ± 32.77^a^ ^,^ ^b^
E/A	0.84 ± 0.3	1.25 ± 0.40^a^	1.09 ± 0.27^a^	0.81 ± 0.29	1.17 ± 0.33^a^	1.10 ± 0.26^a^
TAPSE (mm)	23.4 ± 2.5	23.9 ± 2.4	23.8 ± 2.4	23.7 ± 2.4	23.3 ± 3.3	22.8 ± 3.0
RS wave (m/s)	21.1 ± 2.5	21.6 ± 2.4	21.7 ± 2.4	21.3 ± 2.5	21.8 ± 2.4	21.6 ± 2.4

*Note:* In this table we reported the study variables of study population at baseline and follow‐up end.

Abbreviations: a, peak *trans*‐mitral flow velocities in late diastole by pulse‐wave Doppler imaging; BMI, body mass index; BSA, body surface area; CRP, C reactive protein; DecE‐time, deceleration time of E wave; E/A, E wave/A wave ratio; E, peak *trans*‐mitral flow velocities in early diastole by pulse‐wave Doppler imaging; Hb1Ac, 1Ac glycated haemoglobin; HDL, high density lipoprotein; HOMA‐IR, homoeostasis model for the assessment of insulin resistance; IL6, interleukin 6; LAD, left atrium diameter; LAV, left atrium volume; LDL, low density lipoprotein; LV, left ventricle; LVEF, left ventricle ejection fraction; LVTDd, left ventricle telediastolic diameter; LVTSd, left ventricle telesystolic diameter; miR, microRNA; RS, right ventricle S wave; SGLT2, sodium glucose transporter 2; SGLT2i, sodium glucose transporter 2 inhibitors; TAPSE, Tricuspid Annular Plane Systolic Excursion; TNFα, tumour necrosis factor alpha; WHR, waist hip ratio.

^a^ *p* < 0.05 versus group 1.

^b^ *p* < 0.05 when comparing group 2 versus group 3.

^c^ For *p* < 0.05 when comparing follow‐up end versus baseline for each cohort of study.

**FIGURE 1:**
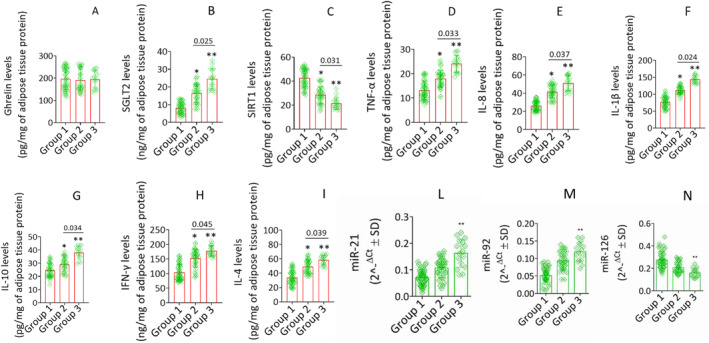
Ghrelin, SGLT2, sirtuins and inflammatory status content in abdominal fat. (A) Ghrelin, (B) SGLT2, (C) SIRT1, (D) TNF‐α, (E) IL‐8, (F) IL‐1β, (G) IL‐10, (H) IFN‐γ and (I) IL‐4 levels in abdominal fat specimens from diabetic obese patients of Group 1 (*n* = 80), Group 2 (*n* = 96) and Group 3 (*n* = 28) assessed by ELISA assay on homogenates. Data are reported as mean ± SD of *n* = 3 independent experiments. IFN‐γ, interferon gamma; IL‐10, interleukin 10; IL‐1β, interleukin 1 beta; IL‐4, interleukin 4; IL‐8, interleukin 8; SGLT2, sodium glucose transporter 2; SIRT1, sirtuin 1; TNF‐α, tumour necrosis alpha. *is for *p* < 0.05 versus Group 1; ** is for *p* < 0.01 versus Group 1.

In contrast, no differences in the serum expression of ghrelin (*p* > 0.05) were recorded among the study cohorts (Table 1). Notably, patients with LVDD (group 3 vs. group 1, and group 2 vs. group 1) displayed the highest values of serum expression of miR‐21 and miR‐92 (*p* < 0.05), and lowest serum expression of miR‐126 (*p* < 0.05; Table 1, Figure 1).

### 3.2 Abdominal Fat Tissue Expression of Ghrelin, SGLT2 Levels, SIRT1, and Inflammatory Markers

No significant changes in ghrelin levels among the studied population were detected (Figure 1A), while tissue SGLT2 expression increased in patients with (group 2 and group 3) versus those without LVDD (group 1). Indeed, patients in group 2 versus group 1 over‐expressed fat tissue SGLT2 (*p* < 0.05), with a greater extent in group 3 as compared to group 1 (*p* < 0.01) and group 2 (Figure 1B).

Then, SIRT1 levels in subjects from group 2 compared to group 1 were significantly downregulated (*p* < 0.05), with a most significant decrease in fat tissue from group 3 compared to all other groups (Figure 1C). Analogously, cytokine abundancy levels were significantly higher (*p* < 0.05) in the patients with a higher stage of LVDD (i.e. group 3 and group 2 vs. group 1), with a greater extent in group 3 as compared to group 1 (*p* < 0.01; Figure 1D–I). Finally, we found that patients with a higher stage of LVDD (group 3 and group 2 vs. group 1) exhibited an increased tissue expression of miR‐21 and miR‐92 (*p* < 0.01), and a reduced tissue expression of miR‐126 (*p* < 0.01; Figure 1L–N).

### 3.3 Effects of Abdominoplasty on the Clinical Parameters at 1 year of Follow‐Up

After the surgical intervention, we reported post‐operative complications in the post‐procedural phase in 9 patients (4.4%): post‐surgical haematomas in 4 patients (44.4%), infections in 3 patients (33.3%), and suspected or confirmed venous thromboembolism in 2 patients (22.2%). At 1‐year follow‐up, all study groups did not differ for clinical characteristics, glucose homoeostasis, insulin resistance, and drug therapy (*p* > 0.05; Table 1, Supporting Information Table S1); more patients in group 3 were under ACE inhibitors and SGLT2i compared to group 2 and group 3 (*p* < 0.05; Table 1, Supporting Information Table S1). Conversely, the patients with LVDD (group 3 and group 2 vs. group 1) had the highest values of LAD, LAV, and septum diameters (*p* < 0.05; Table 1). Specifically, patients with the worst stage of LVDD (group 3 vs. group 2) had the highest values of LAD, LAV, and septum diameters (*p* < 0.05, Table 1). Patients with LVDD (group 3 and group 2 vs. group 1) also had higher E/A values (*p* < 0.05, Table 1). Finally, patients in group 3 versus those in group 2 and versus those in group 1 had significantly lower DecE‐time values (*p* < 0.05, Table 1). Notably, group 1 of patients showed after abdominoplasty (follow‐up end vs. baseline data), a significant reduction in BMI, heart rate, WHR, HOMA‐IR, insulin, cholesterol, LDL, triglycerides, and creatinine values (*p* < 0.05). Table 1. Regarding echocardiographic parameters, the abdominoplastic surgery caused in this cohort of patients the significant reduction in intima‐media thickness, LAD, septum and posterior wall diameters, LV mass, and LV mass/BSA (*p* < 0.05). Table 1.

Group 2 of patients showed after abdominoplasty (follow‐up end vs. baseline data), a significant reduction in BMI, heart rate, WHR, HOMA‐IR, insulin, cholesterol, LDL, triglycerides, and creatinine values (*p* < 0.05). Table 1. Regarding echocardiographic parameters, the abdominoplastic surgery caused in this cohort of patients the significant reduction in intima‐media thickness, LAD, septum and posterior wall diameters, LV mass, and LV mass/BSA (*p* < 0.05). Table 1. In group 3 of patients with worse diastolic function, we found that the abdominoplastics caused a significant reduction in BMI, heart rate, WHR, HOMA‐IR, LDL, creatinine, and Hb1Ac values at the follow‐up end (*p* < 0.05). Table 1. In this cohort of patients, we found a significant reduction in intima‐media thickness, septum, and posterior wall diameters (*p* < 0.05). Table 1. In the supplementary file, we report the data of T2DM obese patients without LVDD who did not undergo abdominoplastic surgery (control group) at baseline and follow‐up. This cohort was compared, at baseline and at the follow‐up end, to the T2DM obese patients without LVDD (group 1) that undergo abdominoplastic. Then, we compared in this cohort the follow‐up end versus baseline data.

### 3.4 Effects of Abdominoplasty on Serum Inflammatory Markers, SGLT2, and miRs Expression

At 1‐year follow‐up, patients with LVDD (group 3 and group 2 vs. group 1) had the highest values of serum inflammatory markers, nitrotyrosine, miR‐21, miR‐92 and SGLT2 (*p* < 0.05). Table 1. Similarly, the patients with a higher stage of LVDD (group 3 vs. group 2) had higher values of serum inflammatory markers, nitrotyrosine, miR‐21, miR‐92, and SGLT2 (*p* < 0.05). Table 1. Notably, patients with LVDD (group 3 and group 2 vs. group 1) had reduced serum levels of miR‐126 (*p* < 0.05, Table 1). We observed a similar expression trend for serum miR‐126 when comparing patients in group 3 versus those in group 2 (*p* < 0.05, Table 1). Moreover, the excision of fat abdominal tissue induced a significant (*p* < 0.05) increase in serum level of miR‐126, and down‐regulation of miR‐21 and miR‐92 at the end of the follow‐up compared to the baseline condition in each group (Table 1). Additionally, patients with the highest level of LVDD had the lowest serum values of miR‐126 and the most elevated serum values of miR‐21 and miR‐92 at the end of follow‐up (*p* < 0.05, Table 1).

Group 1 of patients at 1 year of follow‐up after abdominoplasty as compared to baseline condition showed the lowest values of inflammatory/oxidative stress markers and of miR‐21 and miR‐92 (*p* < 0.05). Table 1. They over‐expressed at 1 year of follow‐up after abdominoplastic surgery the miR‐126 (0 < 0.05). Table 1. Group 2 of patients at 1 year of follow‐up after abdominoplasty as compared to baseline condition showed the lowest values of inflammatory/oxidative stress markers, miR‐21, miR‐92, and SGLT2 (*p* < 0.05). Table 1. These patients over‐expressed the miR‐126 (*p* < 0.05). Table 1. Regarding the bio‐humoral markers, the abdominoplastic caused in group 3 of patients (follow‐up end vs. baseline data) the significant reduction of TNFα and the significant increase of miR‐126 (*p* < 0.05). Table 1.

### 3.5 Effects of Abdominoplasty on Ghrelin Expression

Excision of the fat abdominal tissue induced a significant increase in serum ghrelin at the end of follow‐up compared to baseline in each group (*p* < 0.05, Table 1). Notably, patients with LVDD (group 3 and group 2 vs. group 1) had the lowest serum expression of ghrelin (*p* < 0.05, Table 1). We also found a significantly reduced serum expression of ghrelin in the patients with a higher stage of LVDD (group 3 vs. group 2) (*p* < 0.05, Table 1). Group 1 of patients at 1 year of follow‐up after abdominoplasty, as compared to baseline condition, over‐expressed serum ghrelin (0 < 0.05). Table 1. The same effect was observed in group 2 and group 3 of patients (*p* < 0.05). Table 1.

### 3.6 Effects of Abdominoplasty on the Normalisation of LV Diastolic Function

One year after the excision of fat abdominal tissue, 12 patients in group 1 had a worsening LV diastolic function; these 12 patients exhibited increased E/E′ values (9 < E/E′ < 14). Thus, they became group 2 at the end of the follow‐up. Similarly, two patients from group 2 had a worsening of the LV diastolic function (E/E′ > 14) and became group 3. Instead, four patients from group 3 had an amelioration of LV diastolic function (9 < E/E′ < 14) and became group 2; 36 patients from groups 2 and 14 from group 3 experienced normalisation of LV diastolic function (E/E′ < 9). Furthermore, at the end of the follow‐up period, we had 118 patients with normal LV diastolic function (E/E′ < 9), and 50 patients reached the study outcome, which was the normalisation of LV diastolic function (E/E′ < 9).

### 3.7 Multivariate Cox Regression Analysis Results: Relation Between the Study Variables and Clinical Study Outcome

**FIGURE 2:**
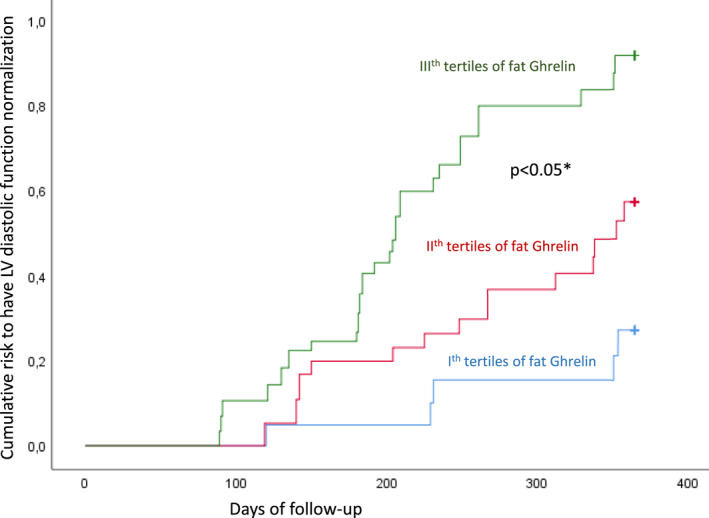
Kaplan curves (lower part) for the cumulative risk to have the normalisation of LV diastolic function at 1 year of follow‐up in study cohorts divided for the different tertiles of fat ghrelin expression. E/E′: E wave/E′ wave ratio. The symbol * is for statistical significant (*p* < 0.05).

We investigated the relationship between study variables and study outcome, which was the normalisation of LV diastolic function (E/E′ < 9) at 1 year via Cox regression analysis. We found that abdominal adipose tissue expression of miR‐126 (HR 1.344, CI 95% 1.126–1.937), and ghrelin (HR 1.123, CI 95% 1.016–1.310) were independent predictors of LV diastolic function normalisation at the end of follow‐up (Table 2). We divided the study population into tertiles according to the expression of ghrelin in the abdominal adipose tissue: patients were divided into 1st tertile (ghrelin ≤ 412 A.U.), 2nd tertile (412 < ghrelin < 662 A.U.), and 3rd tertile (ghrelin < 662 A.U) of abdominal adipose tissue ghrelin expression. Thus, the population was stratified according to tertiles of adipose tissue ghrelin expression in the Kaplan curves for the cumulative risk of LV diastolic function normalisation at 1 year in the study (Figure 2). We observed that the cumulative risk of reaching LV diastolic function normalisation at 1 year was significantly higher in patients with the highest value of ghrelin expression in the abdominal adipose tissue (Figure 2).

**TABLE 2:** Cox regression analysis for risk factors predictive of study endpoint at 1 year follow‐up.

Risk factors	Univariate analysis			Multivariate analysis		
	HR	CI 95%	*p* value	HR	CI 95%	*p* value
Systolic arterial pressure	1.004	0.980–1.028	0.764			
Ghrelin	1.021	1.014–1.027	0.001*	1.123	1.016–1.310	0.001*
miR126	1.466	1.235–1.925	0.029*	1.344	1.126–1.937	0.037*
CRP	0.705	0.297–1.671	0.427			
SIRT1	0.928	0.837–0.989	0.050*	1.006	0.856–1.183	0.938
SGLT2i	0.979	0.491–1.952	0.952			

*Note:* The symbol * is for statistical significant (*p* < 0.05).

Abbreviations: CI, confidence interval; CRP, C reactive protein; CRP: SGLT2i, sodium glucose transporter 2 inhibitors; E/E′, E wave/E′ wave ratio; HR, Hazard ratio; miR‐126, microRNA 126; SIRT1, sirtuin 1.

## 4 Discussion

In the present study, we investigated the correlation between ghrelin expression and LV diastolic function in a cohort of T2DM obese patients treated by abdominal fat tissue excision. Patients with LVDD had over‐inflammation and higher levels of SGLT2 at baseline, a lower expression of SIRT1 and miR‐126, and an increased expression of miR‐21, and miR‐92. After surgical fat excision (and dietary regimen), the patients experienced an amelioration of LV diastolic function with a significant reduction of serum inflammatory molecules, SGLT2, miR‐21, and miR‐92, and up‐regulation of miR‐126 and serum ghrelin. We observed the highest serum ghrelin levels (and lowest serum SGLT2 levels) in patients with normal LV diastolic function at the end of follow‐up and in those with lower LVDD (group 2 vs. group 3; *p* < 0.05). Consequently, a higher rate of patients in group 3 (higher degree of LVDD) as compared to the other study groups were under ACE inhibitors and SGLT2i (*p* < 0.05). Finally, the highest tissue expression of miR‐126 (HR 1.344), and ghrelin (HR 1.123) could increase the probability of LV diastolic function normalisation rate at 1 year of follow‐up after surgical fat excision.

The miR‐21 and miR‐92 are over‐expressed in T2DM patients with diastolic HF [15]. Empagliflozin inhibits the SGLT2‐mediated pathways and induces the down‐regulation of the miR‐21 and miR‐92 and ameliorates endothelial function [15]. Empagliflozin reduces the risk of cardiovascular death or hospitalisation in patients with HF and preserves the ejection fraction regardless of the presence or absence of diabetes [11]. In our study, T2DM obese patients with improved LV diastolic function had reduced levels of miR‐21 and miR‐92 and inflammatory markers, and higher levels of serum miR‐126 and ghrelin. In animal models, ghrelin was associated with protective cardiovascular effects, rescuing SIRT1 and reducing inflammatory/oxidative stress via miR‐126 expression [14]. These ghrelin‐induced effects might lead to the normalisation of LVDD via downregulation of inflammatory burden, SGLT2, miR‐21, and miR‐92, and over‐expression of miR‐126 [14, 15]. In rats with chronic HF, the chronic subcutaneous administration of ghrelin improved LV function and attenuated the development of LV remodelling and cardiac cachexia [16]. Inflammation could also cause advanced oxidation and heart dysfunction in obese patients [20], and the SIRT1 is directly involved in cardiac remodelling during pathological adaptive conditions [21, 22]. Intriguingly, SGLT2i reduced the risk of HF worsening or cardiovascular death among patients with preserved ejection fraction beyond the condition of diabetes [11]. Notably, in our cohorts of T2DM obese patients, we found that the fat surgical excision might induce the ghrelin‐mediated cardioprotective effects (including the amelioration of LV diastolic function) via the SGLT2 down‐regulation and the specific modulation of miR expression. Ghrelin is a natural endogenous ligand of the growth hormone (GH) receptor and a potent stimulant for releasing GH [23]; its receptors are widely distributed in cardiovascular and heart tissues [24]. The exogenous administration of ghrelin improved endothelial function by reducing inflammatory/oxidative stress and inhibiting myocardial cell apoptosis [25, 26, 27]. Besides, ghrelin suppresses high glucose‐induced apoptosis of endothelial cells by activating PI3K/Akt and reducing reactive oxygen species generation [28], inflammation [29], and migration [30]. In patients with chronic HF, ghrelin improves ventricular remodelling [31, 32], decreases cardiac injury [31, 32], and reduces infarct size [33] by rescuing the SIRT1 activity via the modulation of miR‐126 [34]. SIRT1 induction showed anti‐inflammatory effects per se in an ob/ob mice model by rescuing miR‐126 expression [35]. In this context, patients with higher ghrelin tissue expression at baseline had a 1.1‐fold higher probability of having a normal LV diastolic function at the end of follow‐up. Of note, among the patients stratified for tertiles by the levels of ghrelin in the fat, those in the third tertile had a significantly higher likelihood of reaching the normalisation of LV diastolic function at the end of follow‐up (*p* < 0.05; Figure 2) compared to the patients in the second and first tertiles. The patients with higher tissue expression of miR‐126 had a 1.3‐fold higher probability of having a normal LV diastolic function at the end of follow‐up. Indeed, miR‐126 is implicated in cardiovascular protection via the modulation of angiogenesis, inflammation, and apoptosis in T2DM patients [36]. Consistently, miR‐126 is expressed at significantly lower levels in the heart of diabetic rats, associated with diastolic HF [37], and is down‐regulated in T2DM patients with LVDD [15]. These data could show the implication of miRs in the modulation of cardiac adaptive processes in humans [38]. The current study presents some limitations. The relatively short duration of follow‐up (12 months) does not allow us to investigate long‐term outcomes. Thus, longer and larger prospective studies are warranted to confirm our findings. We did not use animal or cellular models to test the clinical results obtained by peripheral blood analysis or by directly analysing samples from abdominal fat tissue biopsy. We did not have data on the acylated and/or non‐acylated ghrelin form; nevertheless, these forms have shown the same binding affinity for their receptor; thus, we do not expect any major difference in the control of the mechanisms responsible for LV diastolic (dys)function [25]. In our population, we had a small percentage of patients under insulin therapy; in these patients, the use of HOMA to assess insulin sensitivity could be a limiting factor [16, 39, 40]. Despite this issue, from clinical and epidemiological studies, a good correlation between estimates of IR derived from HOMA and other tests, such as the euglycaemic clamp and the minimal model, has been reported [16, 39, 40]. Finally, we would suggest that changes in anthropometric and lifestyle factors could play a role in the relationship between insulin resistance and T2DM. On the other hand, the absence of significant differences in body weight and other clinical characteristics (BMI, WHR, etc.), glucose homoeostasis, and insulin resistance parameters (insulin levels and HOMA‐IR) reduces this possible study bias.

## 5 Conclusions

Our data indicate that at 12 months of follow‐up, the patients showed a significant decrease in BMI and WHR post abdominoplastic surgery and diet. This significant weight loss is linked to a significant reduction in the inflammatory burden and SGLT2 levels, miRs modulation, and increased ghrelin expression. These induced effects could ameliorate LVDD, and at follow‐up end we had 50 patients (24.5%) with normalisation of LVDD. Notably, compared with those with LVDD, the patients without LVDD had the highest serum levels of ghrelin and miR‐126 and the lowest serum values of miR‐21, miR‐92, and SGLT2 at the end of the 12‐month follow‐up. Furthermore, the higher abdominal fat tissue expression of miR‐126 and ghrelin could increase of 1.3 and 1.1 folds, respectively, the normalisation of LV diastolic function. Thus, we concluded that the abdominal fat excision and diet could significantly ameliorate the LVDD via increasing serum ghrelin and reducing serum inflammatory molecules and SGLT2 levels in type 2 diabetic obese patients.

## Author Contributions

**Celestino Sardu:** study design, statistical analysis, manuscript writing and editing. **Nunzia D'Onofrio:** study experiments, immunohistochemistry, and figures. **Maria Consiglia Trotta:** study experiments, immunohistochemistry, and figures. **Giovanni Francesco Nicoletti:** surgical interventions. **Carla Contaldi:** study methodology and echocardiographic evaluation. **Giuseppe Pacileo:** study methodology and echocardiographic evaluation. **Matilde Sbriscia:** study methodology and echocardiographic evaluation. **Giovanbattista D'Amico:** data collection. **Maria Luisa Balestrieri:** study revision. **Giuseppe Signoriello:** statistical analysis. **Raffaele Marfella**: study revision and editing. **Giuseppe Paolisso:** study revision and editing.

## Ethics Statement

The ethical committee of participating institutions approved the study; the patients provided informed consent to participate in the study.

## Consent

All authors give full consent for publication.

## Conflicts of Interest

The authors declare no conflicts of interest.

### Peer Review

The peer review history for this article is available at https://www.webofscience.com/api/gateway/wos/peer-review/10.1002/dmrr.70049.

## Supporting information

Tables S1

Figure S1

## Data Availability

Data available on request.
