# Supplementary material for: Could Ghrelin Expression Regulate Diastolic Cardiac Function in Type 2 Diabetic Obese Patients?
Source: Diabetes Metab Res Rev. 2025 May 5;41(4):e70049. doi: 10.1002/dmrr.70049 (PMC12051781; doi:10.1002/dmrr.70049)
Supplement: Supplementary file 1 — Tables S1 [file DMRR-41-e70049-s001.docx]

**Supplementary table 1.** In this table we reported the drug therapy at baseline and at follow-up end in study cohorts of type 2 diabetes mellitus (T2DM) obese patients tat undergo the intervention of abdominoplasty: patients with normal left ventricle diastolic function (group 1) vs. those with left ventricle diastolic dysfunction (group 2 and group 3). ACE is angiotensin converting enzyme; ARS is angiotensin renin system; ASA: acetylsalicylic acid; DPP4i: dipeptidyl peptidase 4 inhibitors; GLP1-RA: Glucagon-like peptide-1 receptor agonists; SGLT2i: sodium glucose transporter 2 inhibitors. *:p<0.05 vs. group 1; # p<0.05 when comparing group 2 vs. group 3.

**Supplementary table 2.** In this table the baseline and follow-up end data, including drug therapy, of patients without left ventricle diastolic dysfunction at baseline that undergo intervention of abdominoplastic surgery (group 1, n 80) vs. those that did not undergo abdominoplastic surgery (control group, n 83). BMI: body mass index; BSA: body surface area; CRP: C reactive protein; DecE-time: deceleration time of E wave; E/A: E wave/A wave ratio; E: peak trans-mitral flow velocities in early diastole by pulse-wave Doppler imaging; a: peak trans-mitral flow velocities in late diastole by pulse-wave Doppler imaging; Hb1Ac: 1Ac glycated hemoglobin; HDL: high density lipoprotein; HOMA-IR: homeostasis model for the assessment of insulin resistance; IL6: interleukin 6; LAD: left atrium diameter; LDL: low density lipoprotein; LAV: left atrium volume; LV: left ventricle; LVEF: left ventricle ejection fraction; LVTDd: left ventricle telediastolic diameter; LVTSd: left ventricle telesystolic diameter; miR: microRNA; RS: right ventricle S wave; SGLT2: sodium glucose transporter 2; SGLT2i: sodium glucose transporter 2 inhibitors; TAPSE: Tricuspid Annular Plane Systolic Excursion; TNFα: tumor necrosis factor alpha; WHR: waist hip ratio; ACE is angiotensin converting enzyme; ARS is angiotensin renin system; ASA: acetylsalicylic acid; DPP4i: dipeptidyl peptidase 4 inhibitors; GLP1-RA: Glucagon-like peptide-1 receptor agonists; SGLT2i: sodium glucose transporter 2 inhibitors. *:p<0.05 vs. group 1; Ŧ for p<0.05 when comparing follow-up end vs. baseline for each cohort of study.

**Supplementary table 3. Cox regression analysis for risk factors predictive of study endpoint (normalization of left ventricle (LV) diastolic function, as E/E’<9) at 1 year follow-up.** In the model A, we included BMI; in model B, we included the WHR; in model C, we included age; in model D, we included the gender; in the model E, we included the HOMA-IR; in the model F, we included the eGFR. miR-126: microRNA 126; CRP: C reactive protein; E/E’: E wave/ E’ wave ratio; CRP: SGLT2i: sodium glucose transporter 2 inhibitors; SIRT1: sirtuin 1; HR: Hazard ratio; CI: confidence interval; BMI (body mass index); WHR: Waist to Hip ratio; HOMA-IR: Homeostatic Model Assessment of Insulin Resistance; eGFR: estimated Glomerular Filtration Rate. The symbol * is for statistical significant (p<0.05).

**Supplementary table 1. Drug therapy at baseline and at follow-up end in study cohorts.**

|  | **BASELINE** |  |  | **1 YEAR OF FOLLOW-UP** |  |  |
| --- | --- | --- | --- | --- | --- | --- |
|  | **Group 1 (n 80)** | **Group 2 (n 96)** | **Group 3 (n 28)** | **Group 1 (n 118)** | **Group 2 (n 74)** | **Group 3 (n 12)** |
| ***Drug therapy*** |  |  |  |  |  |  |
| ACE inhibitors (%) | 34 (42.5) | 50 (52.1) | 16 (57.1) | 48 (40.7) | 36 (48.6) | 8 (66.6) *^,#^ |
| ARS blockers (%) | 18 (22.5) | 24 (25.0) | 8 (28.6) | 24 (20.3) | 18 (24.3) | 4 (33.3) |
| Calcium channels blockers (%) | 8 (10.0) | 10 (10.4) | 2 (7.1) | 12 (10.2) | 8 (10.8) | 2 (16.6) |
| Beta blockers, (%) | 18 (22.5) | 22 (22.9) | 6 (21.4) | 26 (22.0) | 18 (24.3) | 4 (33.3) |
| Statin (%) | 34 (42.5) | 42 (43.7) | 12 (42.8) | 50 (42.4) | 32 (43.2) | 6 (50.0) |
| Thiazides (%) | 22 (27.5) | 28 (29.2) | 8 (28.6) | 32 (27.1) | 22 (29.7) | 4 (33.3) |
| Loop diuretics (%) | 12 (15.0) | 16 (16.7) | 4 (14.3) | 18 (15.2) | 12 (16.2) | 2 (16.6) |
| ASA, (%) | 22 (27.5) | 24 (25.0) | 8 (28.6) | 34 (28.8) | 22 (29.7) | 4 (33.3) |
| Metformin (%) | 46 (57.5) | 56 (58.3) | 18 (64.3) | 70 (59.3) | 44 (59.4) | 6 (50.0) |
| SGLT2i | 14 (17.5) | 18 (18.8) | 4 (14.3) | 20 (16.9) | 15 (20.2) | 4 (33.3) *^,#^ |
| Incretins  DPP4i  GLP1-RA | 6 (7.5)  2 (2.5) | 8 (8.3)  2 (2.1) | 2 (7.1)  0 (0) | 18 (15.2)  4 (3.4) | 12 (16.2)  2 (2.7) | 2 (16.7)  0 (0) |
| Sulfaniluree | 22 (27.5) | 28 (29.2) | 8 (28.6) | 32 (27.1) | 22 (29.7) | 4 (33.3) |
| Insulin, (%) | 8 (10.0) | 10 (10.4) | 2 (7.1) | 14 (11.9) | 10 (13.5) | 2 (16.6) |

**Supplementary table 2. Baseline and follow-up end data, including drug therapy, of patients without left ventricle diastolic dysfunction at baseline that undergo abdominoplastic surgery (group 1, n 80) vs. those that did not undergo abdominoplastic surgery (control group, n 83).**

|  | **BASELINE** |  | **1 YEAR OF FOLLOW-UP** |  |
| --- | --- | --- | --- | --- |
|  | **Group 1 (n 80)** | **Control group**  **(n 83)** | **Group 1 (n 118)** | **Control group**  **(n 57)** |
| **Study variables** |  |  |  |  |
| ***Clinical variables*** |  |  |  |  |
| Age | 42.5±8.4 | 43.0±9.3 | 43.5±8.7 | 44.19.2 |
| Male (%) | 22 (27.5) | 25 (30.1) | 34 (28.8) | 18 (31.6) |
| BMI (Kg/m^2^) | 34.5±4.04 | 34.3±4.0 | 32.7±3.35 ^Ŧ^ | 34.2±3.7* |
| Systolic arterial pressure (mmHg) | 128.4±10.1 | 129.0±9.0 | 127.5±10.8 | 129.6±9.5 |
| Diastolic arterial pressure (mmHg) | 83.4±8.9 | 83.7±9.0 | 81.3±8.3 | 84.2±9.3 |
| Heart rate (beats for minute) | 76.1±9.0 | 76.4±9.7 | 68.5±9.5 ^Ŧ^ | 74.6±11.0* |
| WHR | 0.91±0.007 | 0.91±0.009 | 0.88±0.003 ^Ŧ^ | 0.91±0.002* |
| HOMA-IR | 3.5±0.98 | 3.5±0.91 | 2.9±0.80 ^Ŧ^ | 3.5±1.20* |
| Insulin (IU/mL) | 37.7±8.46 | 38.3±8.51 | 32.4±7.35 ^Ŧ^ | 38.5±8.58 |
| Glucose (mmol/L) | 6.10± 0.84 | 6.12± 0.89 | 6.06± 0.69 | 6.12± 0.91* |
| Cholesterol (mmol/L) | 4.67±0.95 | 4.68±0.97 | 4.29±0.78 ^Ŧ^ | 4.70±0.98 |
| HDL(mmol/L) | 1.88±0.41 | 1.88±0.54 | 1.91±0.49 | 1.85±0.43* |
| LDL(mmol/L) | 3.16±0.37 | 3.17±0.22 | 2.82±0.63 ^Ŧ^ | 3.19±0.31* |
| Triglycerides(mmol/L) | 1.89±0.44 | 1.92±0.53 | 1.81±0.53 ^Ŧ^ | 1.91±0.50 |
| Creatinine (mmol/L) | 98.6±4.4 | 98.8±4.6 | 92.4±3.8 ^Ŧ^ | 99.1±5.0* |
| Hb1Ac (%) | 6.4±0.05 | 6.4±0.07 | 6.3±0.06 | 6.4±0.17 |
| ***Biohumoral markers*** |  |  |  |  |
| CRP (mmol/L) | 0.79± 0.32 | 0.80±0.33 | 0.57± 0.06 ^Ŧ^ | 0.82±0.41* |
| IL6(pg/ml) | 4.18±0.43 | 4.18±0.40 | 3.89±0.26 ^Ŧ^ | 4.25±0.12* |
| TNFα (pg/ml) | 7.44±0.16 | 7.48±0.20 | 5.86±0.22 ^Ŧ^ | 7.53±0.30* |
| Nitrotyrosine (nmol/l) | 4.39±0.21 | 4.44±0.27 | 3.52±0.63 ^Ŧ^ | 4.61±0.66* |
| miR-21x10^2^, A.U. | 0.71±0.12 | 0.72±0.09 | 0.45±0.33 ^Ŧ^ | 0.84±0.10* |
| miR-92 x10^2^, A.U. | 0.66±0.12 | 0.68±0.15 | 0.28±0.06 ^Ŧ^ | 0.72±0.25* |
| miR-126 x10^2^, A.U. | 1.61±0.25 | 1.60±0.19 | 3.23±0.80 ^Ŧ^ | 1.72±0.49* |
| SGLT2 | 1.03±0.52 | 1.08±0.60 | 0.94±0.42 | 1.11±0.60* |
| Ghrelin | 299.58±81.64 | 303.35±81.83 | 1060.49±354.79 ^Ŧ^ | 352.64±73.8* |
|  |  |  |  |  |
| ***Echocardiographic parameters*** |  |  |  |  |
| Intima-media thickness | 1.01±0.15 | 1.02±0.17 | 0.84±0.17 ^Ŧ^ | 1.0±0.11* |
| LVTDd (mm) | 55.4±4.2 | 55.2±5.0 | 54.3±4.0 | 55.0±4.8 |
| LVTSd (mm) | 32.5±4.2 | 31.3±4.0 | 31.8±3.2 | 31.6±3.4 |
| LVEF (%) | 56.2±6.5 | 57.0±5.7 | 57.8±6.6 | 56.8±6.0 |
| LAD (mm) | 39.1±4.3 | 39.3±4.4 | 35.1±4.3 ^Ŧ^ | 40.3±5.2* |
| LAV (ml/mq) | 28.9±3.14 | 28.3±3.22 | 28.1±3.05 | 29.7±3.90* |
| Septum (mm) | 12.2±2.1 | 12.1±2.0 | 9.8±1.8 ^Ŧ^ | 12.3±2.5* |
| Posterior wall (mm) | 10.1±1.1 | 9.9±1.3 | 9.1±1.0 ^Ŧ^ | 10.2±1.8* |
| LV mass (g) | 208.7±73.4 | 203.9±74.5 | 143.1±43.8 ^Ŧ^ | 215.8±56.3* |
| LV mass/BSA (g/m^2^) | 95.54±34.9 | 94.43±33.3 | 66.31±21.8^Ŧ^ | 96.28±30.1* |
| DecE-time (m/s) | 186.95±26.75 | 187.7±28.65 | 183.10±41.13 | 184.2±41.3 |
| E/A | 0.84±0.3 | 0.86±0.3 | 0.81±0.29 | 0.82±0.29 |
| TAPSE (mm) | 23.4±2.5 | 23.2±2.2 | 23.7±2.4 | 22.1±2.7 |
| RS wave (m/s) | 21.1±2.5 | 21.7±2.8 | 21.3±2.5 | 21.0±3.0 |
|  |  |  |  |  |
| ***Drug therapy*** |  |  |  |  |
| ACE inhibitors (%) | 34 (42.5) | 33 (39.8) | 48 (40.7) | 24 (42.1) |
| ARS blockers (%) | 18 (22.5) | 17 (20.5) | 24 (20.3) | 13 (22.8) |
| Calcium channels blockers (%) | 8 (10.0) | 10 (12.0) | 12 (10.2) | 8 (14.0) |
| Beta blockers, (%) | 18 (22.5) | 17 (20.5) | 26 (22.0) | 13 (22.8) |
| Statin (%) | 34 (42.5) | 35 (42.2) | 50 (42.4) | 25 (43.9) |
| Thiazides (%) | 22 (27.5) | 20 (24.1) | 32 (27.1) | 17 (29.8) |
| Loop diuretics (%) | 12 (15.0) | 14 (16.9) | 18 (15.2) | 11 (19.3) |
| ASA, (%) | 22 (27.5) | 24 (28.9) | 34 (28.8) | 18 (31.6) |
| Metformin (%) | 46 (57.5) | 49 (59.0) | 70 (59.3) | 35 (61.4) |
| SGLT2i | 14 (17.5) | 15 (18.1) | 20 (16.9) | 11 (19.3) |
| Incretins  DPP4i  GLP1-RA | 6 (7.5)  2 (2.5) | 7 (8.4)  3 (3.6) | 18 (15.2)  4 (3.4) | 9 (15.8)  3 (5.2) |
| Sulfaniluree | 22 (27.5) | 26 (31.3) | 32 (27.1) | 20 (35.1) |
| Insulin, (%) | 8 (10.0) | 9 (10.8) | 14 (11.9) | 7 (12.3) |

**Supplementary table 3. Cox regression analysis for risk factors predictive of study endpoint (normalization of left ventricle (LV) diastolic function, as E/E’<9) at 1 year follow-up, including new risk factors.**

| **A)** |  | **UNIVARIATE ANALYSIS** |  |  | **MULTIVARIATE ANALYSIS** |  |
| --- | --- | --- | --- | --- | --- | --- |
| **Risk factors** | **HR** | **CI 95%** | **p value** | **HR** | **CI 95%** | **p value** |
| Systolic arterial pressure | 1.004 | 0.980-1.028 | 0.764 |  |  |  |
| Ghrelin | 1.021 | 1.014-1.027 | 0.001* | 1.107 | 1.035-1.184 | 0.003* |
| miR126 | 1.466 | 1.235-1.925 | 0.029* | 1.316 | 0.149-1.671 | 0.130 |
| CRP | 0.705 | 0.297-1.671 | 0.427 |  |  |  |
| SIRT1 | 0.928 | 0.837-0.989 | 0.050* | 1.570 | 0.492-5.010 | 0.446 |
| SGLT2i | 0.979 | 0.491-1.952 | 0.952 |  |  |  |
| BMI | 1.035 | 0.989-1.082 | 0.136 | 1.020 | 0.974-1.070 | 0.389 |
| **B)** |  | **UNIVARIATE ANALYSIS** |  |  | **MULTIVARIATE ANALYSIS** |  |
| **Risk factors** | **HR** | **CI 95%** | **p value** | **HR** | **CI 95%** | **p value** |
| Systolic arterial pressure | 1.004 | 0.980-1.028 | 0.764 |  |  |  |
| Ghrelin | 1.021 | 1.014-1.027 | 0.001* | 1.020 | 1.005-1.080 | 0.027* |
| miR126 | 1.466 | 1.235-1.925 | 0.029* | 0.832 | 0.157-2.410 | 0.140 |
| CRP | 0.705 | 0.297-1.671 | 0.427 |  |  |  |
| SIRT1 | 0.928 | 0.837-0.989 | 0.050* | 1.077 | 0.324-3.576 | 0.904 |
| SGLT2i | 0.979 | 0.491-1.952 | 0.952 |  |  |  |
| WHR | 0.025 | 0.0001-10.182 | 0.818 |  |  |  |
| **C)** |  | **UNIVARIATE ANALYSIS** |  |  | **MULTIVARIATE ANALYSIS** |  |
| **Risk factors** | **HR** | **CI 95%** | **p value** | **HR** | **CI 95%** | **p value** |
| Systolic arterial pressure | 1.004 | 0.980-1.028 | 0.764 |  |  |  |
| Ghrelin | 1.021 | 1.014-1.027 | 0.001* | 1.034 | 1.009-1.060 | 0.008* |
| miR126 | 1.466 | 1.235-1.925 | 0.029* | 0.940 | 0.181-4.877 | 0.942 |
| CRP | 0.705 | 0.297-1.671 | 0.427 |  |  |  |
| SIRT1 | 0.928 | 0.837-0.989 | 0.050* | 1.215 | 0.533-2.902 | 0.274 |
| SGLT2i | 0.979 | 0.491-1.952 | 0.952 |  |  |  |
| Age | 0.995 | 0.969-1.022 | 0.704 |  |  |  |
| **D)** |  | **UNIVARIATE ANALYSIS** |  |  | **MULTIVARIATE ANALYSIS** |  |
| **Risk factors** | **HR** | **CI 95%** | **p value** | **HR** | **CI 95%** | **p value** |
| Systolic arterial pressure | 1.004 | 0.980-1.028 | 0.764 |  |  |  |
| Ghrelin | 1.021 | 1.014-1.027 | 0.001* | 1.087 | 1.007-1.184 | 0.005* |
| miR126 | 1.466 | 1.235-1.925 | 0.029* | 1.218 | 1.104-1.674 | 0.050* |
| CRP | 0.705 | 0.297-1.671 | 0.427 |  |  |  |
| SIRT1 | 0.928 | 0.837-0.989 | 0.050* | 1.116 | 0.334-3.723 | 0.859 |
| SGLT2i | 0.979 | 0.491-1.952 | 0.952 |  |  |  |
| Gender (male) | 0.922 | 0.506-1.680 | 0.790 |  |  |  |
| **E)** |  | **UNIVARIATE ANALYSIS** |  |  | **MULTIVARIATE ANALYSIS** |  |
| **Risk factors** | **HR** | **CI 95%** | **p value** | **HR** | **CI 95%** | **p value** |
| Systolic arterial pressure | 1.004 | 0.980-1.028 | 0.764 |  |  |  |
| Ghrelin | 1.021 | 1.014-1.027 | 0.001* | 1.011 | 1.008-1.070 | 0.020* |
| miR126 | 1.466 | 1.235-1.925 | 0.029* | 1.227 | 0.986-1.624 | 0.322 |
| CRP | 0.705 | 0.297-1.671 | 0.427 |  |  |  |
| SIRT1 | 0.928 | 0.837-0.989 | 0.050* | 0.693 | 0.190-2.522 | 0.578 |
| SGLT2i | 0.979 | 0.491-1.952 | 0.952 |  |  |  |
| HOMA-IR | 0.989 | 0.971-1.007 | 0.232 |  |  |  |
| **F)** |  | **UNIVARIATE ANALYSIS** |  |  | **MULTIVARIATE ANALYSIS** |  |
| **Risk factors** | **HR** | **CI 95%** | **p value** | **HR** | **CI 95%** | **p value** |
| Systolic arterial pressure | 1.004 | 0.980-1.028 | 0.764 |  |  |  |
| Ghrelin | 1.021 | 1.014-1.027 | 0.001* | 1.076 | 1.020-1.108 | 0.014* |
| miR126 | 1.466 | 1.235-1.925 | 0.029* | 1.140 | 1.060-1.643 | 0.041* |
| CRP | 0.705 | 0.297-1.671 | 0.427 |  |  |  |
| SIRT1 | 0.928 | 0.837-0.989 | 0.050* | 0.893 | 0.267-2.983 | 0.854 |
| SGLT2i | 0.979 | 0.491-1.952 | 0.952 |  |  |  |
| eGFR | 0.490 | 0.096-2.501 | 0.391 |  |  |  |
