# Supplementary material for: Could Ghrelin Expression Regulate Diastolic Cardiac Function in Type 2 Diabetic Obese Patients?
Source: Diabetes Metab Res Rev. 2025 May 5;41(4):e70049. doi: 10.1002/dmrr.70049 (PMC12051781; doi:10.1002/dmrr.70049)
Supplement: Supplementary file 2 — Figure S1 [file DMRR-41-e70049-s002.pdf]

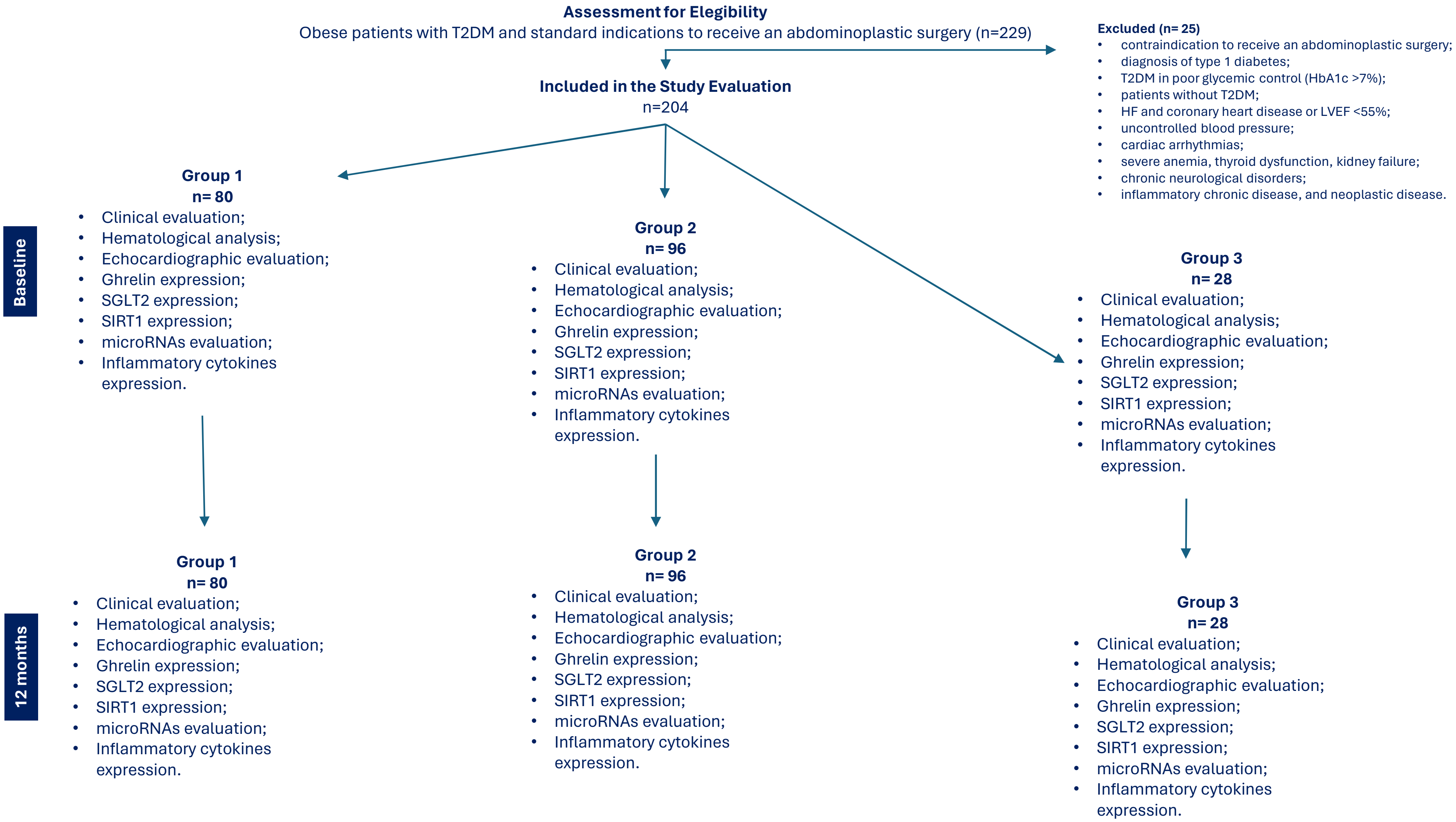

PRIMARY STUDY ENDPOINT: normalization of LV diastolic function at 1 year of follow-up.

SECONDARY STUDY ENDPOINT: serum values of inflammatory markers, SGLT2, miR-21, miR-92, and miR-126 in group 1 vs. group 2 vs. group 3) at 1 year of follow-up.
